# Teaching artificial intelligence through drug–drug interaction clustering analysis: Integrating project-based learning and large language models

**DOI:** 10.1371/journal.pcbi.1014236

**Published:** 2026-05-13

**Authors:** Ji Lv, Guixia Liu, Changjun Zhou

**Affiliations:** 1 School of Computer Science and Technology, Zhejiang Normal University, Jinhua, China; 2 College of Computer Science and Technology, Jilin University, Changchun, China; 3 Key Laboratory of Symbolic Computation and Knowledge Engineering of Ministry of Education, Jilin University, Changchun, China; Montreal, CANADA

## Abstract

In recent years, artificial intelligence (AI) has increasingly influenced daily life and scientific research. Traditionally, AI-related courses have targeted computer science majors, while systematic instructional opportunities for early-stage undergraduates from non-computing backgrounds remain limited. To bridge this gap, we developed an AI course that integrates project-based learning with large language models (LLMs). Specifically, we designed four progressive assignments based on our research project (i.e., drug–drug interaction network clustering analysis). The course does not require prior knowledge of pharmacology or programming. Instead, LLMs are used as assistive tools to support programming, data analysis, and result interpretation. Students engage in a complete workflow, including data curation, algorithm implementation, and critical evaluation of results. Preliminary feedback shows that this approach supports the development of problem-solving skills and increases student engagement. This study provides a framework for integrating LLMs into project-based learning. We believe that this teaching proposal will be valuable and inspiring for educators seeking to design or enrich similar courses.

## 1. Introduction

Historically, artificial intelligence (AI) education has been primarily designed for computer science and related disciplines [1,2]. Such courses typically emphasize algorithmic principles, and programming implementation [3]. While this paradigm has been effective in training technical specialists, it poses substantial challenges for students without a computer science background. These challenges include high barriers to entry, limited opportunities for hands-on practice, and difficulties in transferring learned concepts to domain-specific problems, all of which hinder the broader adoption of AI across disciplines.

The rapid development of Large language models (LLMs) has begun to reshape this landscape [4]. LLMs (e.g., ChatGPT, DeepSeek, Gemini) have lowered the technical threshold for AI use, catalyzing their widespread adoption in fields beyond computer science, including science, engineering, humanities, and arts [5,6]. In response, universities worldwide have introduced general AI education courses designed for non-computer science students to improve AI literacy [7–10]. Despite these efforts, many existing programs remain focus mainly on tool demonstrations or conceptual overviews, with limited emphasis on practical application. As a result, students may develop a basic understanding of AI concepts but still struggle to apply them to real-world problems.

Project-based learning (PBL) is an effective approach to promote active learning and problem-solving skills and has been widely used in science and engineering education [11,12]. In PBL, students learn by working on real-world problems and engaging in hands-on tasks. Bioinformatics provides a particularly suitable context for PBL [13–15], as it involves real biological datasets, algorithm implementation, and progressive scientific discovery. However, the interdisciplinary nature and technical complexity of bioinformatics often pose substantial challenges for students lacking programming experience and biological background.

**Fig 1:**
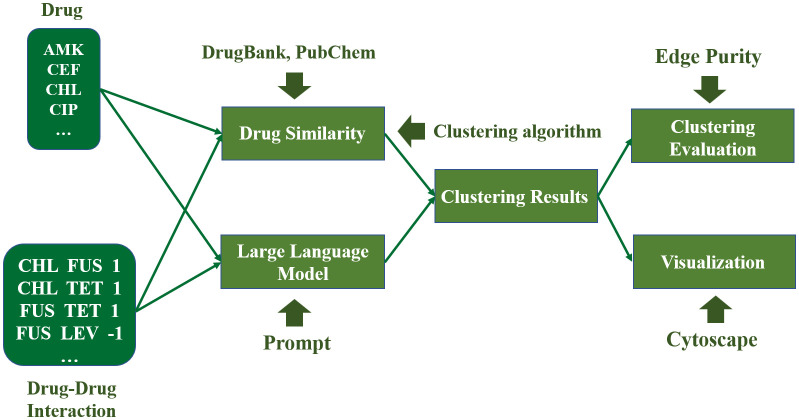
A workflow for DDI network clustering analysis.

LLMs provide new opportunities to address these challenges. LLMs can assist students with coding and reasoning [16,17]. In this study, we propose a general AI education framework and course syllabus (i.e., Artificial Intelligence: Practice and Applications) designed specifically for first-year undergraduate students without prior training in biology or computer science. In this course, LLMs are used not only to lower the entry barrier to programming but also to support human–machine collaborative problem solving. Therefore, this course is not intended to be a project-based bioinformatics course. Instead, it uses bioinformatics case studies as an entry point to provide a realistic application scenario for general AI education. Importantly, the course integrates outcomes from our National Natural Science Foundation-funded research projects into case-based teaching materials [18–26]. Such integration not only helps students connect course content with current scientific research but also allows instructors to design more meaningful and engaging assignments based on their domain expertise [27]. In addition, this approach can also enhance student engagement and provide opportunities for research-informed learning. We use drug–drug interaction (DDI) network clustering analysis as the central instructional case and implement a PBL framework based on this task (Fig 1). Compared with other commonly used bioinformatics teaching examples (e.g., sequence analysis or gene expression analysis), this task places less emphasis on complex biological mechanisms and is therefore more accessible to beginners. It allows students without prior background to focus on AI technologies (e.g., data analysis, clustering methods). The course consists of four interconnected assignments, each focusing on a distinct aspect of DDI network clustering. Through these assignments, students perform clustering analyses based on various drug information to predict unknown DDI and infer their potential mechanisms of action. The resulting predictions can then be evaluated against published scientific literature, allowing students to validate their findings. In this process, students effectively assume the role of drug scientists and engage in the analysis and exploration of DDI networks.

In the following sections, we describe the course content and provide a detailed overview of the four assignments, including their learning objectives and the core problems that students are expected to address. We also discuss possible extensions and optional challenges associated with each assignment. The proposed teaching materials were implemented at Zhejiang Normal University and evaluated using data collected from 85 first-year undergraduate students in science and engineering programs who were enrolled in the course.

## 2. Course content

**Fig 2:**
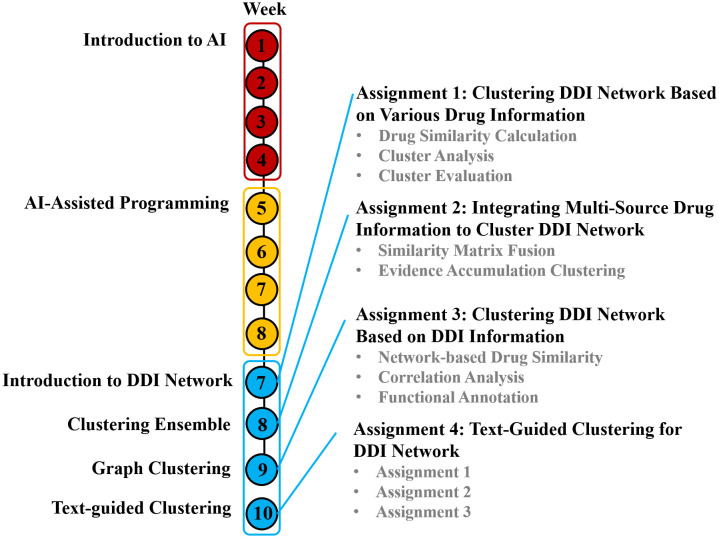
The course schedule of the Artificial Intelligence: Practice and Applications course.

The course is organized into three teaching modules, each lasting approximately four weeks (Fig 2). In the first module, we provide a systematic introduction to fundamental AI concepts, the evolution of AI, and the practical use of AI tools (e.g., ChatGPT, Gemini). This module aims to help students understand model principles, application scenarios, and ethical considerations. The second module focuses on cultivating essential computational skills. Students are introduced to the Python programming environment (i.e., Anaconda, Spyder), basic syntax, and commonly used libraries (e.g., NumPy, Pandas, Matplotlib, Scikit-learn, RDKit, NetworkX). In addition, AI-assisted programming tools (e.g., GitHub Copilot, ChatGPT Code Interpreter) are incorporated into hands-on sessions. Through guided practice, this module allows students to focus on algorithmic logic and problem-solving rather than relying solely on manual coding. The final module integrates theoretical knowledge with practical skills through a PBL approach. Students first acquire relevant theoretical knowledge through lectures and then complete a series of corresponding assignments. During this process, teachers provide structured but limited guidance. At the early stage, instructors introduce key concepts, demonstrate example workflows, and guide students in using LLMs. As the course progresses, the level of guidance is gradually reduced, and students are encouraged to solve problems independently. In this setting, teachers act as facilitators. Although LLMs are introduced as assistive tools, clear boundaries are defined between student responsibilities and AI assistance. Students are expected to understand and decompose the problem, design the analysis workflow, and evaluate the outputs. LLMs can be used to assist with programming, support debugging, and provide suggestions. Importantly, students are required to critically assess AI-generated results rather than accept them directly. In some cases, incorrect or incomplete outputs from LLMs are used as learning opportunities to help students identify errors and refine their approaches.

Designing assignments for LLM-assisted PBL requires careful consideration from instructors. Many questions that are readily available on the internet or in public repositories may already be included in the training data of LLMs. In such cases, students can obtain answers simply by entering the questions into LLMs, without engaging in meaningful reasoning or critical thinking. Therefore, assignments need to be carefully designed to require multi-step analysis and problem decomposition, so that tasks cannot be solved through simple prompts alone. Recently, Zhao and colleagues [28] developed a benchmark consisting of 500 chemistry questions derived from challenging exam problems and recent scientific literature. These questions were designed to require reasoning and problem decomposition, thereby enabling the evaluation of the chemical reasoning ability of LLMs. Similarly, our assignments were adapted from recent bioinformatics studies [18–26] rather than from publicly available exercise collections. Although this approach may increase the preparation workload for instructors, it encourages students to analyze and decompose problems rather than directly obtaining answers from LLMs.

There are four assignments in this study, designed to support a progressive learning process (Fig 2). At the beginning of the course, many students were not familiar with programming or bioinformatics concepts. Therefore, they are guided to perform DDI network clustering analysis using LLMs. In Assignment 1, students learn how to retrieve drug-related data and compute drug similarities, which helps them understand the data and the problem background. In Assignment 2, students learn how to integrate various drug information and observe how this affects clustering results, highlighting the importance of data integration. In Assignment 3, students explore network-based similarity and analyze relationships between network-based similarity and pharmacological similarity. At this stage, they begin to connect computational results with underlying biological meaning. In Assignment 4, students perform clustering tasks by prompt engineering and compare AI-generated results with those obtained from traditional clustering methods. This stage encourages students to reflect on the strengths and limitations of LLMs. This step-by-step assignment design enables students to gradually progress from basic data processing to more advanced tasks involving data integration, interpretation, and critical analysis.

Each assignment typically consists of 3–4 required questions. In addition, optional questions are provided to offer opportunities for bonus credit. These optional tasks often involve extended analyses, alternative algorithms, or exploratory investigations. For each assignment, students are required to submit a report, including the steps of analysis, results, visualization, and corresponding interpretations. Although the assignments are based on recent bioinformatics studies, the biological concepts are simplified to ensure accessibility for students from diverse backgrounds. The primary goal of this course is to cultivate general AI skills such as data analysis, AI-assisted programming, and result interpretation. Therefore, instructors do not need deep expertise in bioinformatics to implement this framework. Detailed course materials, including slides, datasets, assignment descriptions, and code resources, are available at http://acdb.plus/DDI/.

## 3. Course assignments

The course consists of four assignments that progressively introduce clustering methods and their applications in DDI network analysis.

### 3.1 Assignment 1: Clustering DDI network based on various drug information

In the first assignment, students are introduced to the fundamental drug-related concepts, including drug information (e.g., chemical structures, mechanisms of action, ATC codes, and bacterial growth curves), adjuvants, and drug combinations (i.e., synergy effect, additive effect, antagonism effect). This assignment guides students to retrieve drug information from public databases, compute drug similarity [29], and apply clustering algorithms (e.g., hierarchical clustering, spectral clustering) to perform clustering analysis on DDI networks.

#### 3.1.1 Learning outcomes.

In this assignment, students are expected to:

- Become familiar with various drug information and retrieve them from public databases.
- Compute drug similarity based on various drug information and perform clustering analysis.
- Examine and evaluate the quality and biological plausibility of the clustering results.

#### 3.1.2 Required questions.

In this assignment, students use a dataset [30] reported by Chandrasekaran and colleagues, which includes 18 antibiotics covering various antibiotic classes (e.g., aminoglycoside, macrolide, quinolone).

The primary objective of this assignment is to cluster these 18 antibiotics based on four drug information (i.e., chemical structure, mechanism of action, bacterial growth curves, and ATC codes), and to explore potential group-group interaction patterns. Before starting the assignment, students are required to collect drug information from public databases (e.g., ACDB [25], AADB [24], PubChem [31], DrugBank [32]) and to install the necessary Python libraries (e.g., Scikit-learn, RDKit, and Networkx), which are used for similarity computation and clustering analysis. The assignment consists of three required questions:

1. Compute structural, pharmacological, phenotypic, and therapeutic similarities between antibiotics based on their chemical structures, mechanisms of action, bacterial growth curves, and ATC codes, respectively.
2. Implement the spectral clustering algorithm, and cluster the 18 antibiotics into 6 clusters based on each drug similarity.
3. Evaluate clustering quality using edge purity [22] and visualize the clustering results using Cytoscape [33].

To reduce the implementation burden, students can use LLMs as coding assistants. For example, students may prompt an LLM with a structured request such as:

*“Write a Python program to compute pairwise chemical-structure similarity and perform spectral clustering using NumPy, RDKit, Pandas, and scikit-learn. The program should (1) read an Excel file to obtain the compound names and SMILES; (2) convert SMILES into MACCS fingerprints using RDKit; (3) calculate pairwise Tanimoto similarity to obtain a similarity matrix; (4) perform spectral clustering on the similarity matrix; and (5) the final clustering are output in Python dictionary format.”*

#### 3.1.3 Optional questions.

As optional extensions, students are encouraged to explore different numbers of clusters to determine the appropriate number of clusters. Students are also encouraged to explore various clustering algorithms and consult the literature or interact with LLMs to explore additional drug information that could be used for clustering analysis.

#### 3.1.4 Summary.

**Fig 3:**
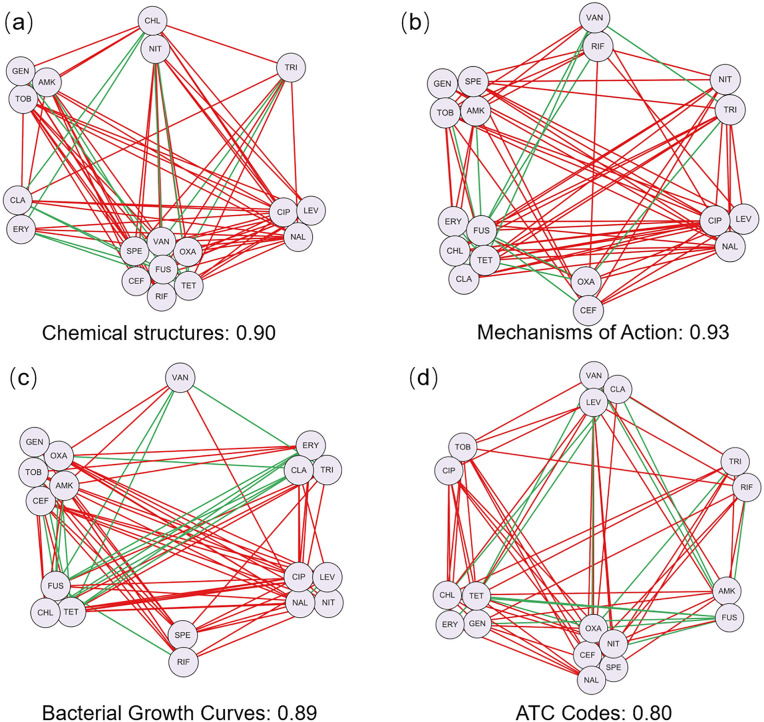
Clustering results of DDI network based on (a) chemical structures, (b) mechanisms of action, (c) bacterial growth curves, and (d) ATC codes. The corresponding edge purity values are 0.90, 0.93, 0.89, and 0.80, respectively.

This assignment is designed to guide students through the complete workflow of drug information retrieval, similarity computation, and DDI network clustering analysis. By clustering the DDI network based on various drug information, students gain insight into group-group interaction patterns among antibiotics. Moreover, the assignment highlights both the shared and distinct characteristics across different drug information (Fig 3), thereby motivating the need for more advanced integrative analyses introduced in subsequent assignments.

### 3.2 Assignment 2: Integrating multi-source drug information to cluster DDI network

**Fig 4:**
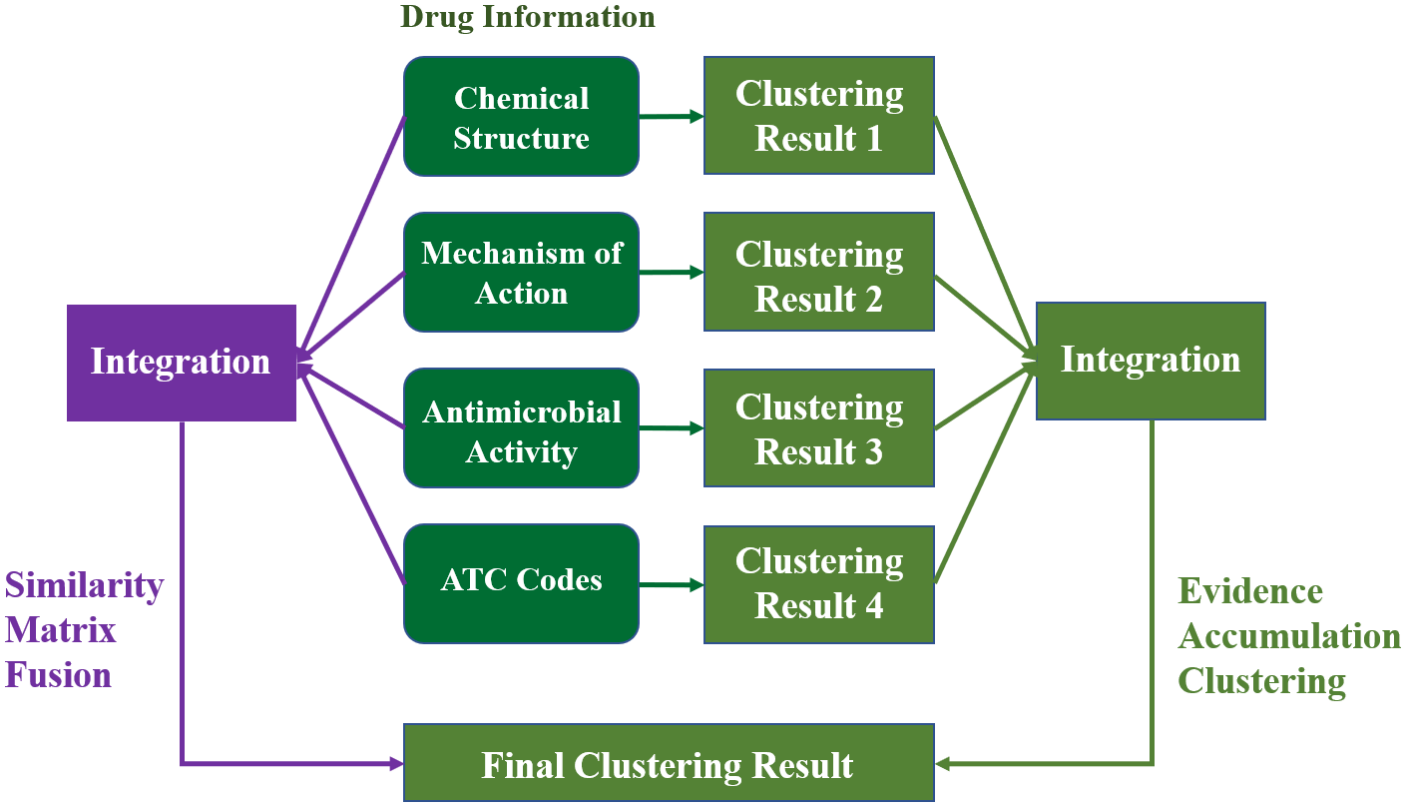
Different clustering ensemble strategies.

In the first assignment, students performed clustering analysis based on four types of drug information. Clustering based on various drug information may led to inconsistent clustering results (Fig 3). To obtain more robust and consistent clustering results, clustering ensemble algorithms (i.e., evidence accumulation clustering [34] and similarity matrix fusion [18]) can be employed. The evidence accumulation clustering algorithm integrates multiple clustering results by constructing a co-association matrix, whereas similarity matrix fusion algorithm combines multiple similarity matrices into a consensus matrix before clustering (Fig 4). Both methods aim to improve the robustness and reliability of clustering results.

#### 3.2.1 Learning outcomes.

In this assignment, students are expected to:

- Implement and apply the evidence accumulation clustering algorithm for DDI network clustering analysis.
- Implement and apply the similarity matrix fusion algorithm to integrate multi-source drug information for DDI network clustering analysis.

#### 3.2.2 Required questions.

In this assignment, students again use the dataset [30] reported by Chandrasekaran and colleagues. The primary objective is to integrate multi-source drug information, thereby obtaining more accurate and robust clustering results. The assignment consists of three required questions:

1. Implement the evidence accumulation clustering algorithm to integrate multiple clustering results obtained in Assignment 1.
2. Implement the similarity matrix fusion algorithm to merge the four drug similarity matrices derived from Assignment 1 into a consensus matrix, followed by clustering.
3. Evaluate the clustering performance of the two ensemble clustering algorithms using edge purity.

#### 3.2.3 Optional questions.

Our analysis demonstrates that integrating multi-source drug information can improve clustering quality. The clustered DDI network exhibits good monochromaticity, in which group-group interactions are predominantly synergistic or antagonistic. Based on these observations, two optional extension tasks are proposed:

1. Which type of drug information is most critical for clustering? Students are encouraged to conduct ablation studies by removing individual drug information (e.g., chemical structure) and evaluating their impact on clustering performance.
2. Can the clustered DDI network be used to predict new DDIs? Students assign three previously unclustered antibiotics (i.e., kanamycin, penicillin G, and roxithromycin) to the clustered DDI network and predict their interactions with the 18 clustered antibiotics, followed by an assessment of predictive performance.

#### 3.2.4 Summary.

**Fig 5:**
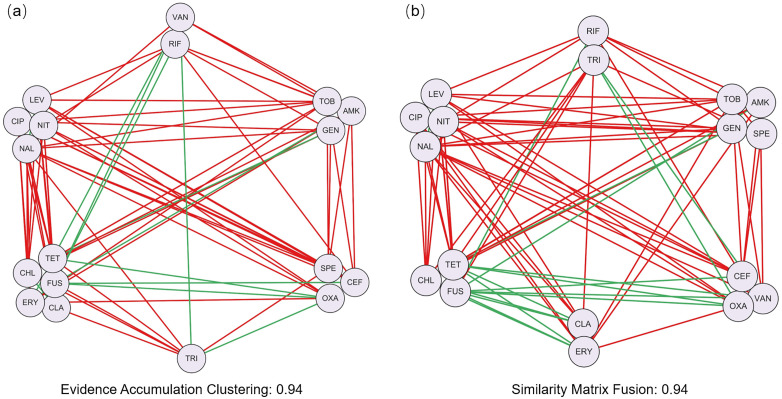
Clustering results of DDI network based on (a) evidence accumulation clustering, (b) similarity matrix fusion. The corresponding edge purity values are 0.94, 0.94, respectively.

In this assignment, students implement two clustering ensemble algorithms to derive a comprehensive and robust antibiotic classification, enabling a more reliable exploration of group-group interaction patterns (Fig 5). The optional questions further highlight the potential of clustered DDI network for predicting unknown DDIs.

### 3.3 Assignment 3: Clustering DDI network based on DDI information

**Fig 6:**
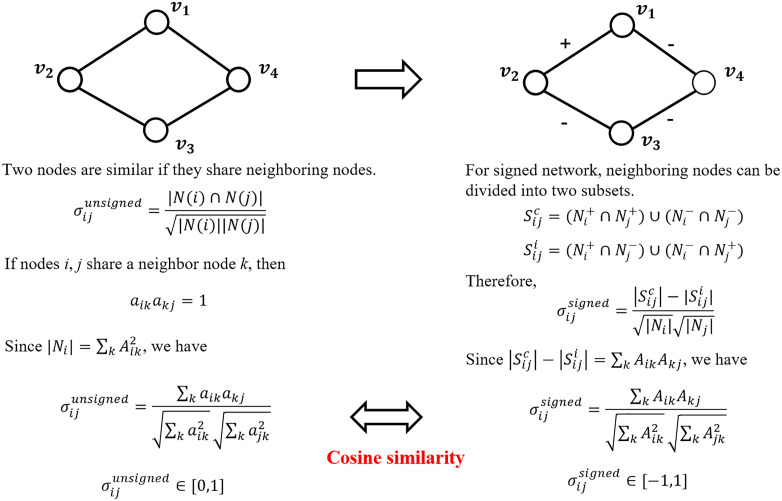
Node similarity in unsigned and signed network.

In the previous assignments, students focused on clustering methods based on drug information (Figs 4 and 5). In this assignment, the focus shifts to the DDI network itself, exploring how DDI information can be directly used for clustering analysis (Fig 6). By analyzing clustered DDI networks, students investigate whether drugs grouped within the same cluster tend to share similar mechanisms of action. In addition, we introduce the concept of adjuvants and discuss the potential applications of this method in inferring mechanisms of action of adjuvants.

#### 3.3.1 Learning outcomes.

In this assignment, students are expected to:

- Compute drug similarity based on DDI information and perform clustering analysis using the network-based similarity.
- Investigate correlations between the network-based similarity and the four previously explored similarity measures.
- Explore potential mechanisms of action of drug inferred from network-based clustering results.

#### 3.3.2 Required questions.

In this assignment, students again use the dataset [30] reported by Chandrasekaran and colleagues. The primary objective is to compute drug similarity directly from DDI network structure and to perform clustering analysis based on this similarity. The assignment consists of three required questions:

1. Compute drug similarity based on DDI information;
2. Investigate correlations between network-based similarity and the four similarities explored in previous assignments (e.g., structural similarity and pharmacological similarity);
3. Explore the properties of the clustered DDI network and discuss its potential application in predicting the mechanisms of action of adjuvants.

#### 3.3.3 Optional questions.

Brochado and colleagues reported a drug combination dataset [35] involving six bacterial strains in 2018. In this optional extension, students compute drug similarity using the DDI information from a single bacterial strain and perform clustering analysis. The resulting clustering is then applied to the remaining strains, and edge purity is used to evaluate clustering performance. Through this exercise, students observe that the network-based similarity is highly sensitive to dataset quality and that noise has a negative effect on the similarity computation. To address this problem, students are encouraged to apply the clustering ensemble strategies introduced in Assignment 2 by integrating multi-species DDI information, thereby obtaining more robust clustering results.

#### 3.3.4 Summary.

**Fig 7:**
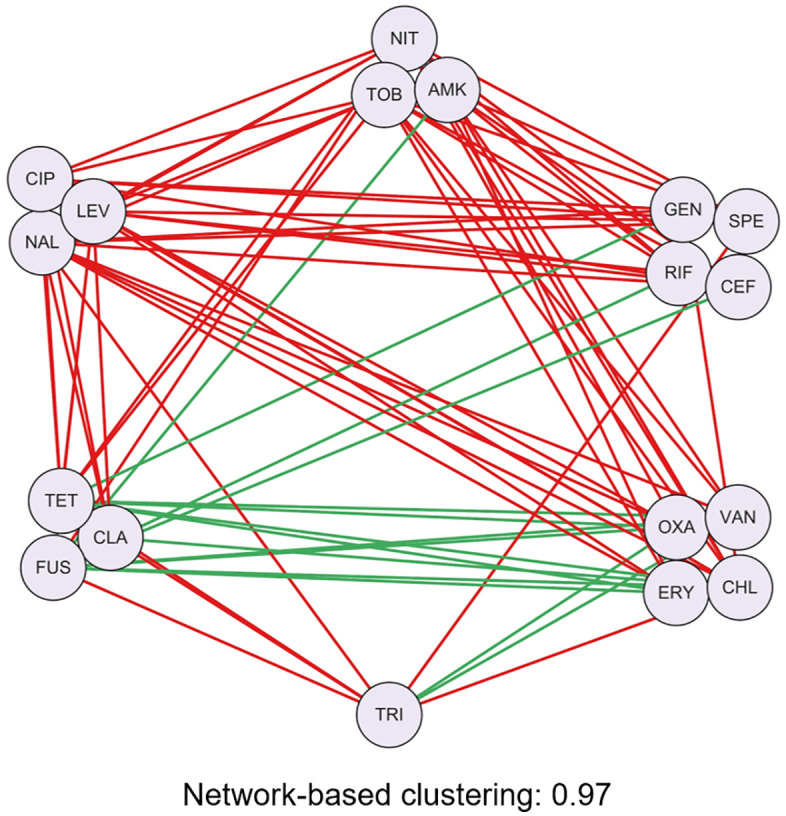
Clustering results of DDI network based on DDI information. The corresponding edge purity value is 0.97.

In this assignment, students compute drug similarity based on DDI network structure and then perform clustering analysis (Fig 7). The network-based similarity shows a strong correlation with pharmacological similarity, highlighting their potential for exploring mechanisms of action of adjuvants. Although the accuracy of this method depends on the quality of the drug combination datasets, the optional tasks guide students to revisit clustering ensemble strategies introduced in Assignment 2 and apply them to mitigate the effects of noise.

### 3.4 Assignment 4: Text-guided clustering for DDI network

In the previous assignments, students performed clustering analyses on DDI networks based on drug information or DDI information using specific clustering methods (e.g., graph clustering, ensemble clustering). However, these methods typically involve labor-intensive processes, including data collection, similarity computation, and clustering analysis. In assignment 4, we investigate the potential of general LLMs (e.g., ChatGPT, DeepSeek) for DDI networks clustering analysis. Instead of computing similarity matrices, students are instructed to use structured prompts to guide LLMs (e.g., ChatGPT, Gemini) to group antibiotics based on drug or DDI information. Representative prompts used in this assignment are as follows:

“*There are 18 antibiotics. Dividing these antibiotics into 6 groups according to their chemical structures. Output in abbreviated and Python dictionary format.*”,

“*This is a dataset of antibiotic combinations. The first and second columns are abbreviations of antibiotics, and the third column is the type of antibiotic combination. Please classify the antibiotics into 6 groups, and make sure the group-group interactions are predominantly either synergy or antagonism effects.*”

Students may refine the prompt through adding constraints or clarifying grouping criteria. The LLM-generated clustering results are then compared with those obtained from traditional clustering methods in Assignments 1–3. To evaluate the results, students analyze the consistency between text-guided clusters and conventional clustering outputs. They are also required to critically assess whether the LLM-generated groupings are biologically meaningful.

#### 3.4.1 Learning outcomes.

In this assignment, students are expected to:

- Design effective prompts to guide LLMs in performing clustering analysis on DDI networks;
- Compare LLM-assisted clustering with traditional clustering methods in terms of clustering quality and computational efficiency;
- Understand the limitations of LLMs (e.g., hallucination and bias) and explore potential strategies.

#### 3.4.2 Required questions.

In this assignment, students are introduced to the 5S strategy‌‌ [36] for prompt design, which emphasizes structured thinking and clarity in human-machine interaction. Students apply the 5S strategy to design prompts that guided LLMs to complete clustering tasks corresponding to Assignments 1–3. To evaluate the performance of LLMs, students compare the clustering results generated by traditional clustering algorithms with those produced by LLMs. The assignment consists of four required questions:

1. Use one type of drug information (e.g., chemical structure, mechanism of action, antimicrobial activity, or ATC codes) to prompt the LLM to perform clustering on the DDI network.
2. Combine multi-source drug information and design prompts that enable LLMs to perform clustering analysis.
3. Guide the LLM to cluster the DDI network using DDI information
4. Compare the performance and efficiency of LLM-assisted clustering with traditional clustering algorithms.

#### 3.4.3 Optional questions.

Although LLMs demonstrate promising clustering performance in the three tasks, their application also introduced new challenges, including hallucinations, reliability, interpretability, and reproducibility. These challenges may negatively impact downstream tasks (e.g., DDI prediction and functional annotation). To handle these challenges, students are encouraged to explore advanced strategies (e.g., ensemble learning [37] and retrieval-augmented generation (RAG) [38]). For example, students may integrate the outputs from multiple LLMs using ensemble learning to improve the clustering stability and accuracy. In addition, RAG can be used to incorporate external knowledge bases, thereby constraining and refining outputs of LLMs.

#### 3.4.4 Summary.

This assignment provides an opportunity for students to perform text-guided clustering and to compare AI-assisted results with those obtained from traditional clustering methods. As shown in Table 1, LLMs can generate reasonable clustering results under carefully designed prompts, without explicitly performing conventional steps (i.e., data collection, similarity computation, and clustering analysis). However, text-guided clustering may suffer from issues such as hallucination and limited reproducibility. This comparison allows students to better understand both the strengths and limitations of LLMs. It also highlights the importance of human interpretation and validation in AI-assisted analysis.

**Table 1:** Comparative performance of text-guided clustering (i.e., ChatGPT 5.2) and traditional clustering methods. Clustering performance is evaluated using edge purity.

	ChatGPT 5.2	Traditional clustering methods
Chemical Structure	0.87	0.90
Mechanism of action	0.89	0.93
Antimicrobial activity	0.88	0.89
ATC code	0.90	0.80
Clustering ensemble	0.92	0.94
Network-based clustering	0.92	0.97

## 4. Discussion

### 4.1 Student evaluation

To evaluate the effectiveness of the course design, students were asked to complete a brief anonymous survey after finishing the four assignments. The survey assessed their perceptions regarding interest levels, perceived difficulty, time requirements and suggestions for future improvements.

**Fig 8:**
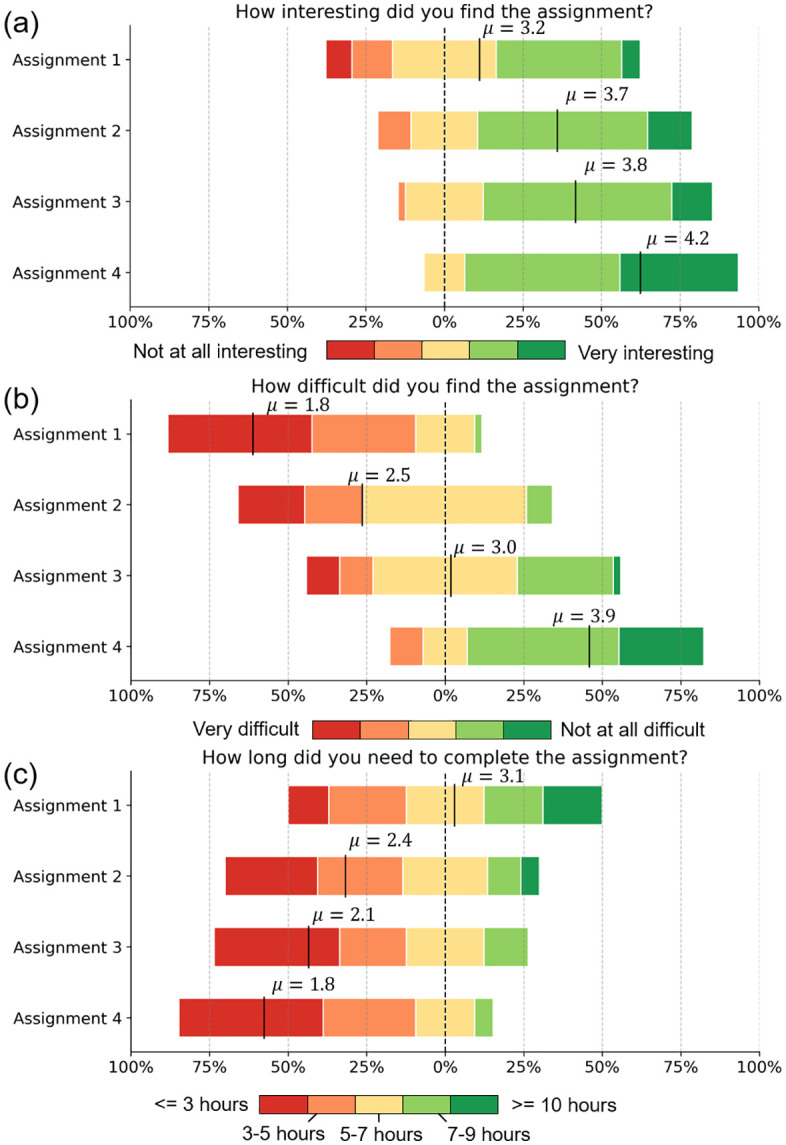
Student feedback. Distributions of student responses regarding assignment **(a)** interestingness, **(b)** difficulty, and **(c)** time required for completion.

As shown in Fig 8a, many students found that these assignments are interesting. Notably, reported interest increased progressively from Assignment 1 to Assignment 4. The initial lack of interest was attributable to student’s unfamiliarity with the research domain. In fact, several open-ended feedbacks indicated that students initially lacked interest because the topic was unfamiliar, but deeper engagement with the research questions led to a gradual increase in interest. These findings suggest that sustained exposure to authentic projects can foster intrinsic motivation, even in students without relevant domain knowledge.

Fig 8b shows students’ perceptions of assignment difficult. Assignments 1 and 2 were rated as the most challenging, which is likely attributable to their substantial programming requirements. For example, Assignment 1 required students to install and use external libraries (e.g., RDKit and networkx) and calculate multiple similarity metrics. Assignment 1 required students to implement similarity matrix fusion and consensus clustering algorithms. In contrast, Assignment 4, which did not involve programming, was rated as the easiest.

Students spent approximately 4–7 hours per assignment on average (Fig 8c). The Assignment 4 required significantly less time than the others, highlighting the efficiency gains associated with LLMs. In open-ended feedback, several students explicitly noted this advantage. In addition, students demonstrated a critical awareness of the limitations of LLM-assisted workflows, particularly the risk of hallucinations. Several students emphasized that careful prompt design was necessary to ensure reliable results. Furthermore, students suggested that integrating retrieval-augmented generation (RAG) techniques could provide more reliable drug-related knowledge, thereby improving clustering purity. These feedbacks suggest that students did not perceive large language models as black-box tools, but instead developed a critical understanding of their strengths and limitations.

In the open-ended section of the survey, the feedback was overwhelmingly positive. Students praised the project-based design, noting that each assignment built incrementally on the previous one. Representative comments included: “These assignments are highly exploratory and closely connected to real-world problems. Although challenging, they are very interesting.” Another student reflected, “As an undergraduate student, completing these assignments gave me a great sense of accomplishment.” Others noted that the debugging process enhanced my understanding of both algorithms and code implementation, and that observing performance improvements after applying similarity matrix fusion methods was particularly rewarding.

Despite these positive outcomes, students also provided constructive criticism. Given that this was the inaugural offering of the course, this feedback is particularly valuable. Criticisms primarily centered on ambiguous assignment instructions and a lack of worked examples and references. For example, “I don’t know how to verify the results,” “More information about this field could be provided,” “Some exercise descriptions could have been clearer.” Despite our intention to encourage self-driven learning, these expectations may have been overly ambitious for first-year students. Therefore, future iterations will offer supplementary learning materials and recorded tutorials for relevant software tools to better support student learning.

### 4.2 Analysis of student AI usage

To understand how students used AI tools during the completion of assignments, we categorized their AI usage behavior into four levels based on their interaction patterns with LLMs:

- **Level 1**: Students treated AI primarily as a search engine and used it to generate simple content.
- **Level 2**: Students learned to write clearer prompts to obtain more accurate or useful outputs from AI.
- **Level 3**: Students treated AI as a thinking partner and complete tasks through iterative dialogue.
- **Level 4**: Students decomposed complex tasks into multiple steps and involve AI throughout the entire workflow.

**Fig 9:**
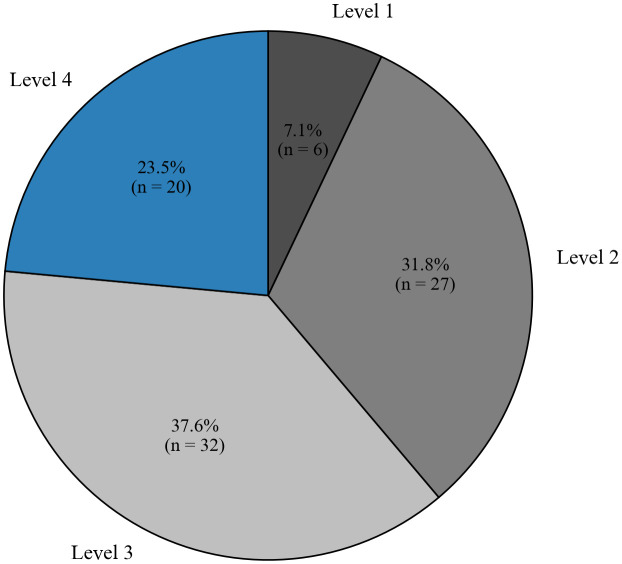
Levels of AI usage observed among students.

As shown in Fig 9, 7.1% of the students (6 out of 85) directly copied the assignments into LLMs without providing further guidance. In these cases, the generated results were often incomplete or inaccurate. In contrast, 23.5% of the students (20 out of 85) can analyze the problems more deeply and decompose them into smaller tasks, which generally resulted in more reliable outputs. Previous studies have shown that LLMs perform well when given explicit instructions for code implementation [39]. However, when tasks require complex reasoning or detailed analysis, LLMs may produce confident but incorrect answers [40]. Therefore, students need to understand the assignments in depth and decompose them into smaller tasks to identify limitations, correct errors, and guide the model toward reliable results.

This requirement changes the nature of effort during learning. Although LLMs can reduce the programming burden for students, they may introduce new forms of cognitive load. Students need to design effective prompts, interpret AI-generated outputs, and verify the correctness of the results. In this sense, the learning process shifts from manual code implementation to task decomposition, human-machine collaboration, and critical evaluation of AI-generated outputs. Therefore, LLM-assisted learning changes the types of skills required during problem-solving.

### 4.3 Limitations

This study has several limitations. First, the course was implemented in a single cohort, and no control group was included. Therefore, it is difficult to directly compare the effectiveness of this approach with traditional teaching methods. Second, the evaluation was based on a single implementation cycle of the course. Long-term effects and year-to-year consistency were not assessed. Future studies could include multiple cohorts to evaluate the stability and generalizability of the teaching framework. Third, the assignments were designed based on a specific task (DDI network clustering). Future work could explore other datasets and tasks to further assess the generality of this approach. Despite these limitations, this study provides a practical framework for integrating LLMs and bioinformatics into PBL and offers insights into AI-assisted education.

## 5. Conclusion

Project-based learning is an effective approach for promoting student engagement and motivation. In this study, we designed and implemented a series of assignments centered on a real-world research problem (i.e., DDI network clustering analysis), allowing first-year science and engineering undergraduates to learn AI through hands-on practice. In this framework, teachers defined the core research questions, while students used LLMs as assistive tools for programming, analysis, and result interpretation. The assignments were structured as progressive units that guided students from basic data processing to more advanced tasks involving integration, interpretation, and critical evaluation. Preliminary feedback suggests that this approach was well received by students. Many students found that the assignments both interesting and challenging. This learning framework not only supported the development of problem-solving skills but also encouraged interest in the interdisciplinary area of AI and biology. Overall, this study provides an initial framework for integrating LLMs into project-based learning, with an emphasis on deep understanding, task decomposition, and critical evaluation rather than direct reliance on AI-generated outputs.
